# The Static Method in X Ray Work

**Published:** 1900-06-23

**Authors:** 


### THE STATIC METHOD IN *X* RAY WORK.

Dr. Arthur Griffiths[1] draws attention to a subject which has been very much neglected in England, namely, the use of static machines as a source of electricity for *x* ray work. He says that a strange apathy has hitherto existed on the part of electricians in this country in regard to the use of static machines as a source of high tension currents for this purpose, although ever since Röntgen's announcement of his discovery such machines have been almost the chief apparatus in use in America. He recommends a Wimshurst machine, which, he says, can not only be bought at a comparatively small cost, but may be made quite easily by anyone having a small amount of knowledge of carpentry. The only apparatus needed in addition is a Jackson's tube, a luminous screen, and a tube holder.

[1] Practitioner, June.

